# 4255 Indiana Clinical and Translational Sciences Institute (CTSI) – CTSA-wide podcast opportunity

**DOI:** 10.1017/cts.2020.274

**Published:** 2020-07-29

**Authors:** Christine Drury, Aaron E. Carroll

**Affiliations:** 1 Indiana University School of Medicine

## Abstract

OBJECTIVES/GOALS:

- The podcasts highlight work from our partners: Indiana University, Purdue University and the University of Notre Dame.
- Our goal is to expand our podcast reach to include work from at least three additional CTSAs, as well as highlighting the benefits of translational research to the public.

METHODS/STUDY POPULATION:

- Aaron E. Carroll, is the director of Education and Workforce Development for the Indiana CTSI and a popular writer covering health, research, and policy for *The New York Times*. He is host of the Indiana CTSI-sponsored *Healthcare Triage* podcasts as well as the Healthcare Triage YouTube show, with 340,000 subscribers. We will leverage his audience and research expertise to grow the Indiana CTSI podcast participation and increase audience engagement.
- We will eventually allow the nation-wide network of CTSAs to pitch guests and shows covering Translational Research, and invite local CTSA leadership or faculty to participate in the podcast.

RESULTS/ANTICIPATED RESULTS:

- Utilizing quantitative analytics, we expect to see a significant increase in podcast downloads and listeners as we expand our offering to other CTSAs, beyond Indiana
- We expect that the CTSA-wide podcast will increase the nationwide level of knowledge and understanding of NCATS, translational research, and its benefits to society and healthcare.
- We anticipate, through this expanded podcast offering, a growing number of community members who are informed and engaged on the topics of translational research, clinical and translational sciences and beyond.

DISCUSSION/SIGNIFICANCE OF IMPACT:

- Podcasts are a convenient, portable, and efficient form of science communication.
- Podcasts also make information personal and offer us an excellent and innovative communications vehicle to spread the word about translational research, as well as the excellent work happening at each of our CTSAs.

